# Transcription Factor ZmNAC20 Improves Drought Resistance by Promoting Stomatal Closure and Activating Expression of Stress-Responsive Genes in Maize

**DOI:** 10.3390/ijms24054712

**Published:** 2023-03-01

**Authors:** Hui Liu, Songbo Song, Mengyao Liu, Yangwei Mu, Ying Li, Yuxin Xuan, Liangjie Niu, Hui Zhang, Wei Wang

**Affiliations:** College of Life Sciences, National Key Laboratory of Wheat and Maize Crop Science, Henan Agricultural University, Zhengzhou 450046, China

**Keywords:** drought resistance, ZmNAC20, transcription factor, stomatal closure, ABA

## Abstract

Drought is a major environmental threat that limits crop growth, development, and productivity worldwide. Improving drought resistance with genetic engineering methods is necessary to tackle global climate change. It is well known that NAC (NAM, ATAF and CUC) transcription factors play a critical role in coping with drought stress in plants. In this study, we identified an NAC transcription factor ZmNAC20, which regulates drought stress response in maize. *ZmNAC20* expression was rapidly upregulated by drought and abscisic acid (ABA). Under drought conditions, the *ZmNAC20*-overexpressing plants had higher relative water content and survival rate than the wild-type maize inbred B104, suggesting that overexpression of *ZmNAC20* improved drought resistance in maize. The detached leaves of *ZmNAC20*-overexpressing plants lost less water than those of wild-type B104 after dehydration. Overexpression of *ZmNAC20* promoted stomatal closure in response to ABA. ZmNAC20 was localized in the nucleus and regulated the expression of many genes involved in drought stress response using RNA-Seq analysis. The study indicated that ZmNAC20 improved drought resistance by promoting stomatal closure and activating the expression of stress-responsible genes in maize. Our findings provide a valuable gene and new clues on improving crop drought resistance.

## 1. Introduction

Drought limits plant growth and reduces crop production worldwide. With global warming, improving drought resistance is of great importance for crop breeding [1,2]. A variety of strategies have evolved to survive under drought conditions. Plants improve drought resistance mainly by promoting stomatal closure, altering root architecture, adjusting the contents of osmotic substances, and increasing the activities of the antioxidant enzymes [3]. When facing drought stress, plant cells sense the stimuli and activate stress response genes to cope with the stress [4]. It has been demonstrated in numerous studies that transcription factors, such as NAC, WRKY, basic leucine zipper (bZIP), homeodomain-leucine zipper (HD-Zip), drought-response elements binding proteins/C-repeat binding factor (DREB/CBF), and MYB, play crucial roles in enhancing drought resistance in plants [5,6,7]. Many stress-responsive genes are activated by transcription factors [6,7]. NAC transcription factors are one of the largest transcription factor families in the plant, with a highly conserved NAC domain at the N-terminal [8]. The name NAC is derived from three transcription factors: NAM (no apical meristem) in *Petunia hybrida*, ATAF1/2 (*Arabidopsis thaliana* activating factor), and CUC2 (cup-shaped cotyledon) in *Arabidopsis thaliana* [9,10,11]. NAC transcription factors are key targets for crop improvement due to their diverse functions in abiotic stress responses [12]. It is found that NAC transcription factors play an important role in regulating plant response to drought [13,14,15,16,17]. NAC can improve plant drought resistance by activating the expression of stress-responsive genes, promoting stomatal closure, increasing osmotic substance content, and so on [8,18].

In maize, previous studies have identified 116 *ZmNACs* genes, and the function of most of them remains unknown [19]. To date, not many *ZmNACs* genes have been characterized as improving drought resistance in maize. Overexpression of *ZmNAC111* can significantly improve the drought resistance of maize, and the natural variation of the *ZmNAC111* promoter is closely related to drought resistance [13]. *ZmNAC33* is upregulated under drought conditions and can be induced by abscisic acid (ABA). Overexpression of *ZmNAC33* in *Arabidopsis* can enhance drought resistance [20]. Natural Antisense Transcripts (NATs) are long-chain noncoding RNAs whose sequences can complement other transcripts. *ZmNAC48* contains one cis NAT and two significant SNPs associated with plant survival rate under drought conditions. Overexpression of *ZmNAC48* improves plant drought resistance, promotes stomatal closure, reduces plant water loss rate, and increases plant survival rate. However, maize with overexpression of cis NAT of *ZmNAC48* shows a higher water loss rate and larger stomatal aperture. Both *ZmNAC48* and its cis NAT are involved in drought stress response. ZmNAC48 promotes stomatal closure, while the cis NAT of *ZmNAC48* negatively regulates stomatal closure by regulating *ZmNAC48* [16]. ZmNAC55 is localized in the nucleus and upregulated under drought stress, salt stress, cold stress, and ABA induction. Overexpression of *ZmNAC55* in *Arabidopsis* significantly improves drought resistance [14]. *ZmNAC49* can be rapidly induced and upregulated by drought stress. Overexpression of *ZmNAC49* in maize results in the decline of stomatal conductance and stomatal density. Further research shows that ZmNAC49 can directly bind to the *ZmMUTE* promoter to inhibit its expression, thereby reducing stomatal density. ZmNAC49 enhances drought resistance mainly by affecting stomatal density [17]. Overexpression of the NAC transcription factor gene *ZmNST3* (*NAC secondary wall thickening promoting factor3*) can significantly increase the drought resistance of maize. ChIP-seq analysis shows that ZmNST3 can directly bind to *CESA5* and *Dynamin-Related Proteins2A (DRP2A)* promoters to activate their expression. ZmNST3 could increase the expression level of genes related to cellulose synthesis in the secondary cell wall [15]. The NAC transcription factor gene *ZmNUT1* (*necrotic upper tips1*) is mainly expressed in the xylem of roots, stems, and leaves. The mutation of *ZmNUT1* leads to abnormal water transportation. One study finds that ZmNUT1 regulates xylem development by directly regulating the expression of the cellulose synthase gene and cysteine proteolytic enzyme gene, thereby affecting water transportation [21]. The natural variation of the noncoding region in *ZmNAC080308* is associated with drought resistance. Overexpression of *ZmNAC080308* in *Arabidopsis* significantly enhances drought resistance [22]. ZmSNAC13 can improve drought resistance by promoting the expression of *PYL9* and *DREB3* [23]. ZmNAC84 improves drought tolerance by directly regulating the expression of *ZmSOD2* [24].

ABA is a key factor that controls drought stress response. Drought stress rapidly induces the synthesis and accumulation of ABA [25]. In the absence of ABA, Serine/threonine protein phosphatase 2C (PP2C) will interact with SnRK2 (SNF1-related protein kinase) and inhibit its activity. When ABA combines with its receptor pyrabactin resistance (PYR)/PYR1-like (PYL)/regulatory components of ABA receptor (RCAR), it can release the inhibition of PP2C on SnRK2. SnRK2 activates transcription factors to control the expression of stress-responsive genes or regulates the activity of the plasma membrane proteins in guard cells to control the cell turgor and stomatal closure [25,26]. In maize, the ABA receptors ZmPYL8, ZmPYL9, and ZmPYL12 facilitate drought resistance in plants [27]. ZmPP2C26 negatively regulates drought resistance by dephosphorylating ZmMAPK3 and ZmMAPK7. The maize *zmpp2c26* mutant shows enhancement of drought resistance with higher root length, root weight, chlorophyll content, and photosynthetic rate compared with the wild type. The *ZmPP2C26* gene generates *ZmPP2C26L* and *ZmPP2C26S* isoforms by alternative splicing. Overexpression of *ZmPP2C26L* and *ZmPP2C26S* significantly decreases drought resistance in *Arabidopsis* and rice [28]. ZmPP2C-A10 functions as a negative regulator of drought resistance. Deletion of an endoplasmic reticulum stress response element (ERSE) in *ZmPP2C-A10* increases drought resistance [29]. ABA signaling is critical for plants to cope with drought stress [25,30]. There are many transcription factors involved in ABA signaling to regulate drought resistance in maize. The BRI1-EMS suppressor 1 (BES1)/brassinazole-resistant 1 (BZR1) transcription factor positively regulates drought resistance by binding to E-box to induce the expression of downstream stress-related genes. Heterologous expression of *ZmBES1/BZR1-5* in transgenic *Arabidopsis thaliana* results in decreased ABA sensitivity, facilitates shoot growth and root development, and enhances drought resistance with lower malondialdehyde (MDA) content and relative electrolyte leakage (REL) under osmotic stress [31]. The expression of *ZmbZIP33* is strongly upregulated by drought and ABA. Overexpression of *ZmbZIP33* causes an accumulation of ABA content and improves drought resistance in *Arabidopsis* [32]. Lateral organ boundaries domain (LBD) proteins are plant-specific transcription factors. ZmLBD5 negatively regulates drought resistance by impairing ABA synthesis [33].

Stomata are essential structures for plants to control gas exchange and water status. Under drought conditions, plants can reduce water loss by adjusting the size of the stomatal aperture. Stomatal closure is regulated by various signaling molecules, such as ABA, ROS, and Ca^2+^ [34]. Several critical factors that regulate stomatal closure in response to drought stress have been characterized in maize. ZmCPK35 (Ca^2+^-dependent protein kinases) and ZmCPK37 (calcium-dependent protein kinases) regulate the drought resistance of maize by regulating the activity of ZmSLAC1 (the S-type anion channel protein) in the guard cells of maize [35]. ZmSLAC1 is specifically expressed in maize guard cells and participates in regulating stomatal closure under drought conditions. *zmslac1* mutant exhibits drought-sensitive phenotype. *ZmCPK35* and *ZmCPK37* are expressed in maize guard cells. ZmCPK35 and ZmCPK37 can interact with ZmSLAC1 on the cytoplasmic membrane and regulate ion channel activity. In the guard cells of *zmslac1* and *zmcpk37* mutants, S-type anion channel currents activated by ABA are significantly inhibited, while overexpression of *ZmCPK35* and *ZmCPK37* could significantly enhance ABA-activating S-type anion channel currents in guard cells [35]. The gene knockout mutant of *zmmpkl1* (mitogen-activated protein kinase) is less sensitive to severe drought. The stomatal aperture of *ZmMPKL1*-overexpressing plants is higher than those of control plants, which leads to faster water loss. The stomatal aperture of the *zmmpkl1* mutant is smaller than that of the control plant. ZmMPKL1 affects the response of plants to drought by regulating ABA homeostasis in plants [36].

In this study, we identified a NAC transcription factor gene *ZmNAC20* in maize. *ZmNAC20* was induced and upregulated by drought stress and ABA. ZmNAC20 was localized in the nucleus and enhanced drought resistance in maize. ZmNAC20 promoted stomatal closure in response to ABA. Our results suggest that ZmNAC20 plays an important role in drought stress response and stomatal movement in maize.

## 2. Results

### 2.1. ZmNAC20 Is Upregulated by Drought Stress and ABA

*ZmNAC20* (*Zm00001eb288360* in the version of Zm-B73-REFERENCE-NAM-5.0, *Zm00001d038221* in version of Zm-B73-REFERENCE-GRAMENE-4.0*, GRMZM2G180328* in version of B73 RefGen_v3), which has been named in the previous study [37], encodes a NAC-type transcription factor. ZmNAC20 belongs to a large number of members of the family in maize [37]. A previous study performed phylogenetic analysis using the amino acid sequences and indicated that the closest two identified homologous genes of *ZmNAC20* (*GRMZM2G180328*) were *Sb09g020750* in *Sorghum bicolor* and *LOC_Os05g34830* in rice [13]. In the maizeGDB database (www.maizegdb.org, accessed on 7 February 2023), the GO annotations indicated that *ZmNAC20* (*Zm00001eb288360*) was involved in multiple processes, including positive regulation of response to water deprivation, positive regulation of response to salt stress, regulation of defense response to fungus, and response to cold (Figure S1). A previous study indicated that *ZmNAC20* was upregulated by treatment with PEG, which could mimic drought stress [37]. We assumed that *ZmNAC20* was likely involved in drought stress response.

**Figure 1:**
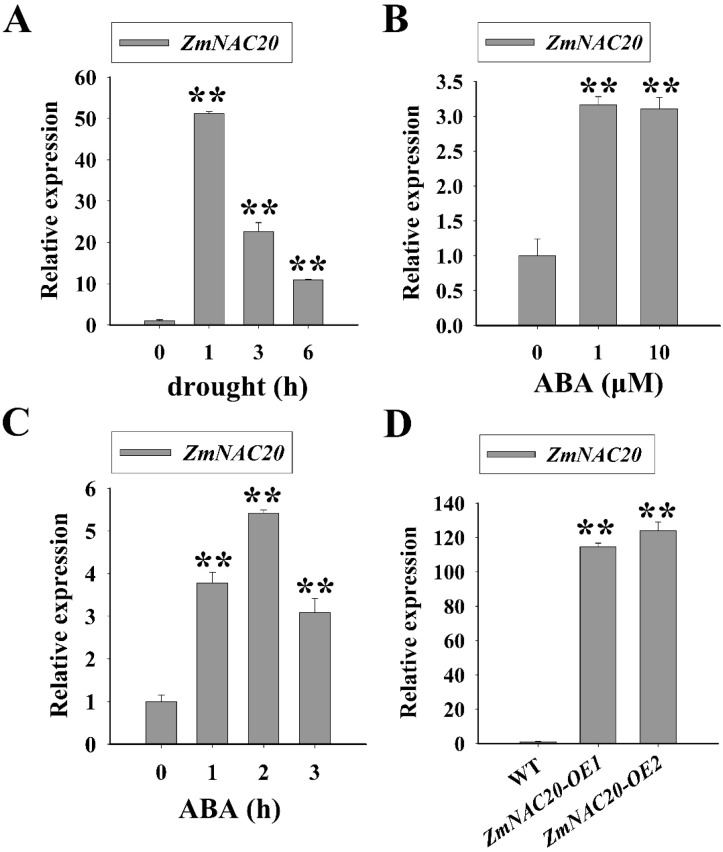
Expression of *ZmNAC20* in response to drought stress and ABA. (**A**) The relative expression level of *ZmNAC20* in response to drought stress. (**B**) The relative expression level of *ZmNAC20* after treatment with different concentrations of ABA. (**C**) The relative expression level of *ZmNAC20* after treatment with 1 μM ABA for 0, 1, 2, and 3 h. (**D**) Relative expression of *ZmNAC20* in *ZmNAC20*-overexpressing transgenic plants. Asterisks indicate significant differences: ** *p* < 0.01 (Student’s *t*-test).

We performed quantitative reverse-transcription PCR (qRT-PCR) to confirm whether *ZmNAC20* expression was regulated by drought stress. When the maize seedlings developed three leaves, the aerial parts were cut and subjected to dehydration stress. The results showed that the expression of *ZmNAC20* was significantly upregulated after dehydration stress (Figure 1A), indicating that drought stress promoted *ZmNAC20* expression. ABA is a critical hormone that regulates drought stress response in plants [25]. The leaves of maize growing for about 12 days were excised and treated with different concentrations of ABA for 1 h. By using qRT-PCR, we found that treatment with ABA elevated the expression level of *ZmNAC20* (Figure 1B). We also examined the expression of *ZmNAC20* treated with 1 μM ABA for 0 h, 1 h, 2 h, and 3 h. *ZmNAC20* was upregulated by ABA at different times (Figure 1C). These results suggested that *ZmNAC20* expression was regulated by both drought stress and ABA.

### 2.2. Overexpression of ZmNAC20 Enhances Drought Resistance in Maize

**Figure 2:**
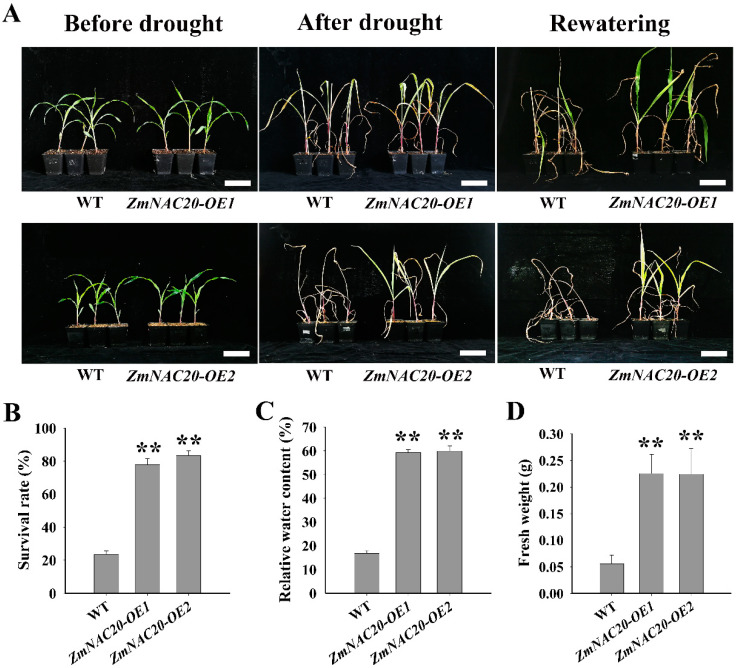
Phenotype of *ZmNAC20*-overexpressing transgenic maize. (**A**) Drought resistance of transgenic maize overexpressing *ZmNAC20*. Images were taken before and after drought stress treatment and rewatering. WT represented the wild-type B104 maize and served as the control. *ZmNAC20-OE1* and *ZmNAC20-OE2* represented the two *ZmNAC20*-overexpressing maize lines. Bars, 5 cm. (**B**) Statistical data for the survival rate of WT, *ZmNAC20-OE1*, and *ZmNAC20-OE2*. The survival rate was obtained from 30 seedings in three independent tests. (**C**) The relative water content of maize leaves under drought stress treatment. The second leaves were cut from drought-treated plants. SE values were calculated from three biological replicates and more than 10 plants were examined in each replicate. (**D**) Fresh weight of maize leaves under drought stress treatment. SD values were calculated from three biological replicates and more than 10 plants were examined in each replicate. Asterisks indicate significant differences: ** *p* < 0.01 (Student’s *t*-test).

To understand whether increased transcript levels of *ZmNAC20* enhanced drought resistance, we generated two transgenic maize which overexpressed *ZmNAC20* under the constitutive promoter *ZmUbiquitin1* (*Ubi*). The two transgenic maize lines were named *ZmNAC20-OE1* and *ZmNAC20-OE2*. Using qRT-PCR assay, we found that the transcript levels of *ZmNAC20* in the two transgenic maize lines were significantly higher than that in the wild type (WT, B104) (Figure 1D). After drought treatment, it was found that the leaves of *ZmNAC20*-overexpression plants were slightly yellow and curly, whereas the leaves of WT plants were severely wilted or even began to die (Figure 2A). After 3 days of rewatering, the leaves of *ZmNAC20*-overexpressing plants gradually expanded and turned green, and the survival rate reached about 80%, much higher than the WT plants (Figure 2A,B). Furthermore, both the relative water content, the fresh weight, and the dry weight of *ZmNAC20*-overexpressing plants were significantly higher than those of WT plants (Figure 2C,D and Figure S2). These results demonstrated that overexpression of *ZmNAC20* improved drought resistance in maize.

### 2.3. Overexpression of ZmNAC20 Promotes Stomatal Closure

**Figure 3:**
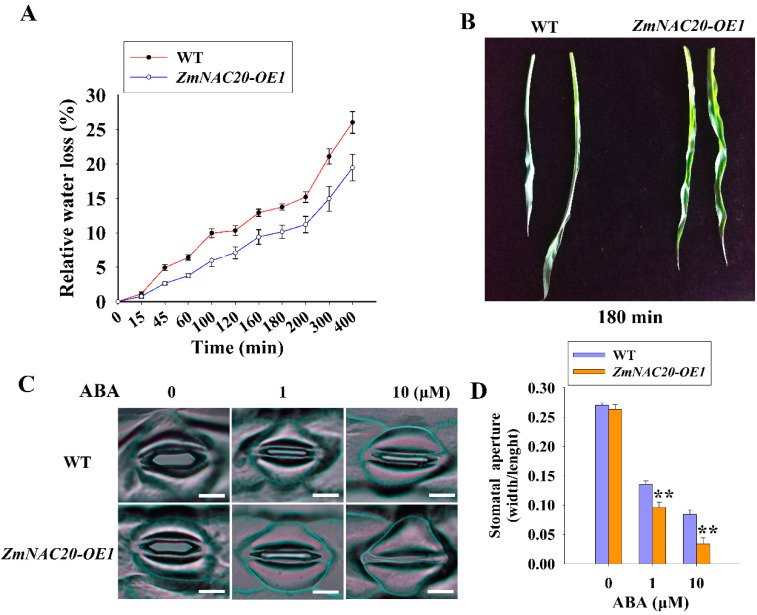
Stomatal aperture analyses of *ZmNAC20*-overexpressing maize. (**A**) Statistics of relative water loss in detached leaves of WT and *ZmNAC20-OE1* after dehydration for indicated times. WT represents the wild-type B104 inbred maize. *ZmNAC20-OE1* represents *ZmNAC20*-overexpressing transgenic maize. (**B**) Photograph of detached leaves after dehydration for 180 min. (**C**) Photograph of the stomatal aperture of detached leaves from WT and *ZmNAC20-OE1* after ABA treatment. Bars, 10 μm (**D**) Statistics of the stomatal aperture of detached leaves after ABA treatment. Asterisks indicate significant differences, ** *p* < 0.01 (Student’s *t*-test).

Stomata are key structures to cope with drought in plants [3,38]. Plants regulated water loss mainly through modulation of the opening and closure of stomata [25]. Rapid stomatal closure is an effective way to reduce water loss and improve the drought resistance of plants [3,25]. The detached leaves of *ZmNAC20*-overexpressing plants showed a slower water loss rate than those of WT plants (Figure 3A). After 180 min of dehydration stress treatment, the leaves of WT had severely curved, whereas the leaves of *ZmNAC20*-overexpressing plants displayed a slightly curled phenotype (Figure 3B). These results suggest that overexpression of *ZmNAC20* could retain more water in leaves and enhance drought resistance in maize. Next, we wondered if ZmNAC20 regulates water loss by modulation of stomatal closure. ABA plays a pivotal role in stomatal closure in response to drought stress, and *ZmNAC20* was upregulated by ABA (Figure 1B,C). We performed the experiment to test whether ZmNAC20 affected the stomatal closure in response to ABA. The stomatal aperture was determined by the ratio of stomatal width to length. It was shown that overexpression of *ZmNAC20* reduced the size of the stomatal aperture under 1 μM and 10 μM ABA treatment (Figure 3C,D). The stomata in *ZmNAC20*-overexpressing plants closed more rapidly than those of WT plants after ABA treatment. Stomatal closure in *ZmNAC20*-overexpressing plants was more sensitive to ABA compared with that of WT plants. These results indicated that ZmNAC20 promoted stomatal closure and impaired water loss, resulting in enhanced drought resistance in maize.

### 2.4. ZmNAC20 Regulates Multiple Biological Pathways under Drought Stress

**Figure 4:**
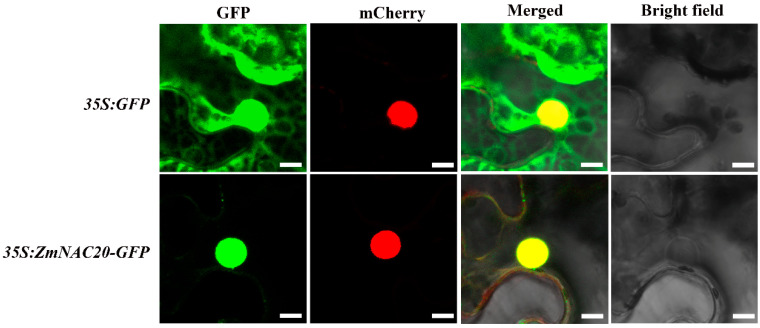
Subcellular localization of ZmNAC20. *35S:H2B-mCherry* is a nuclear localization marker. Either *35S:ZmNAC20-GFP* or the empty vector *35S:GFP* and *35S:H2B-mCherry* were co-transfected into leaves of *Nicotiana benthamiana*. The fluorescence of leaves was imaged using a confocal microscope. The merged signals of GFP and mCherry showed yellow color, suggesting ZmNAC20 was localized in the nucleus. Bars, 10 μm.

Because ZmNAC20 was a NAC transcription factor, we assumed that ZmNAC20 might localize in the nucleus. To test the hypothesis, *p35S:ZmNAC20-GFP* and the nucleus localized construct *35S:H2B-mCherry* were together transformed into leaves of *Nicotiana benthamiana* by agrobacterium-mediated infiltration. The *ZmNAC20-GFP* signal was colocalized with the *H2B-mCherry* signal; a strong yellow fluorescence signal was only detected in the nucleus, whereas the signals of the control construct *35S:GFP* were detected throughout the cell (Figure 4). These results indicated that ZmNAC20 was localized in the nucleus.

**Figure 5:**
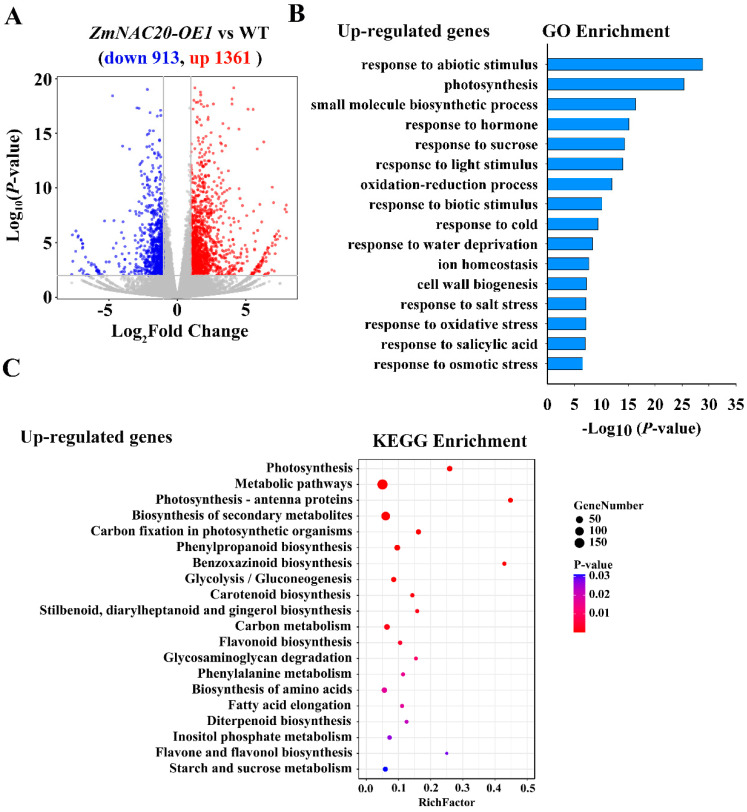
ZmNAC20 regulates multiple signaling under drought stress by RNA-seq analyses. (**A**) Differential gene expression detected by DESeq2. A twofold change in gene expression levels between *ZmNAC20-OE1* and wild-type B104 (WT) with a q-value (adjusted *p*-value) cutoff of 0.05 was considered as a differential expression. A total of 1361 genes were upregulated and 913 genes were downregulated in *ZmNAC20-OE1*. (**B**) GO analyses of upregulated genes in *ZmNAC20-OE1*. (**C**) KEGG analyses of upregulated genes in *ZmNAC20-OE1*.

To uncover the molecular pathways regulated by ZmNAC20 under drought stress, we performed RNA-seq analysis using the detached leaves of *ZmNAC20-OE1* and WT treated with dehydration stress for 3 h. Three biological repeats of the WT (WT-A, WT-B, WT-C) and *ZmNAC20-OE1* (*OE1-A*, *OE1-B*, *OE1-C*) were used for RNA-seq analyses. Twofold gene transcript levels with the adjusted *p-value* (*q* value) cutoff of 0.05 between *ZmNAC20-OE1* and WT was used as the criterion for differential expression. Compared with WT, 1361 genes were upregulated and 913 genes were downregulated in *ZmNAC20-OE1* (Figure 5A, Tables S1 and S2). To reveal ZmNAC20-mediated pathways, we performed Gene Ontology (GO) enrichment analysis and KEGG pathway enrichment analysis. GO analysis showed that the 1361 upregulated genes were mainly involved in multiple biological processes, including responses to abiotic stimulus, photosynthesis, hormone, cold, water deprivation, salt stress, and osmotic stress (Figure 5B). The 913 downregulated genes were mainly involved in the processes of RNA modification, ribosome biogenesis, ribosome assembly, rRNA processing, protein import into the nucleus, and translation (Figure S3). KEGG enrichment analysis indicated that the upregulated genes participated in the pathways of photosynthesis, biosynthesis of secondary metabolites, carbon fixation in photosynthetic organisms, benzoxazinoid biosynthesis, carotenoid biosynthesis, carbon metabolism, and flavonoid biosynthesis (Figure 5C). The downregulated genes were involved in the pathways of ribosome biogenesis in eukaryotes monoterpenoid biosynthesis, tryptophan metabolism, and linoleic acid metabolism (Figure S4). These results suggested that ZmNAC20 regulated multiple biological pathways under drought stress in maize.

### 2.5. ZmNAC20 Regulates the Expression of Stress-Responsive Genes

**Figure 6:**
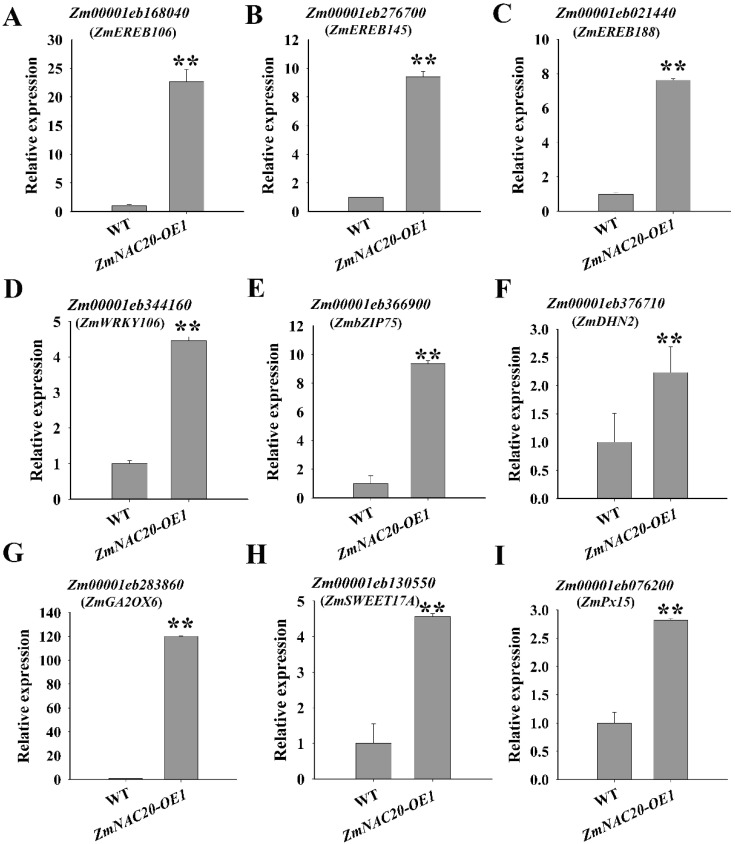
(**A**–**I**) Expression of genes involved in drought stress response in *ZmNAC20-overexpressing* seedlings under drought stress. The seedlings were treated by dehydration for 3 h. SD values were calculated from three biological replicates Asterisks indicate significant differences: ** *p* < 0.01 (Student’s *t*-test), ns indicates no significance.

**Figure 7:**
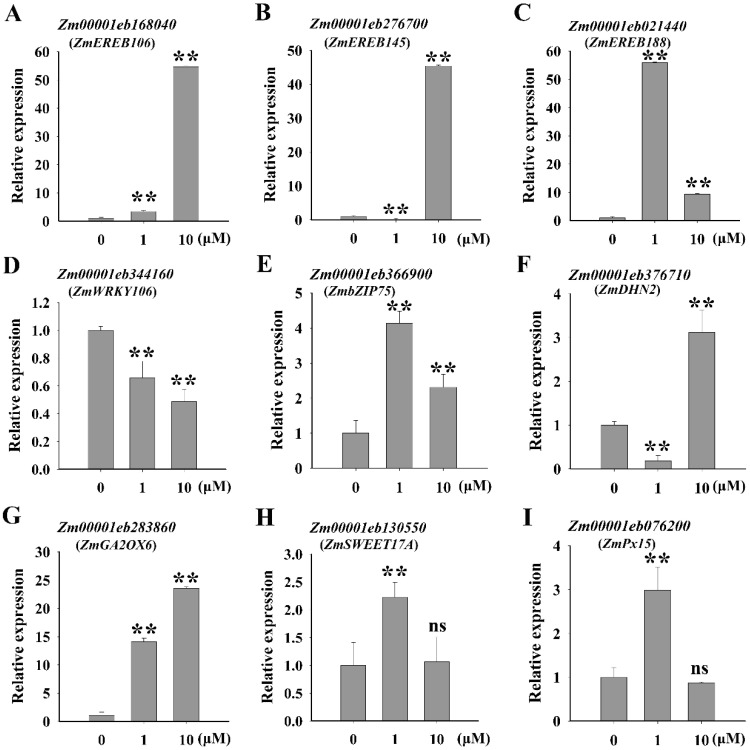
(**A**–**I**) Expression of genes involved in drought stress response by treatment with ABA. The wild-type B104 was treated with different concentrations of ABA for 1 h. SD values were calculated from three biological replicates. Asterisks indicate significant differences: ** *p* < 0.01 (Student’s *t*-test).

RNA-seq analysis revealed that overexpression of *ZmNAC20* affected the expression of many genes. We selected 9 upregulated genes involved in drought stress response for detailed analysis. The gene symbol name used in this study referred to the maizeGDB database (www.maizegdb.org, accessed on 7 February 2023). It is well known that the transcription factors, such as APETALA2/Ethylene Responsive Element Binding Protein (AP2/EREBP), WRKY, and bZIP, play key roles in drought stress response [7,39,40,41]. *Zm00001eb168040* (*ZmEREB106*), *Zm00001eb276700* (*ZmEREB145*), and *Zm00001eb021440* (*ZmEREB188*) are putative AP2/EREBP transcription factor genes. *Zm00001eb344160* (*ZmWRKY106*) is a transcription factor that includes a WRKY DNA-binding domain. *Zm00001eb366900* (*ZmbZIP75*) is a putative bZIP transcription factor. Overexpression of *ZmNAC20* promoted the expression of the four genes by qRT-PCR (Figure 6). After treatment with ABA, *ZmEREB106*, *ZmEREB145*, *ZmEREB188*, and *ZmbZIP75* were upregulated by ABA, whereas *ZmWRKY106* was downregulated by ABA (Figure 7).

Dehydrin is a well-known protein which accumulates massively under drought stress and helps plants improve resistance to stresses [42]. Previous studies have demonstrated many dehydrins involved in drought resistance improvement [43]. For example, *Medicago truncatula MtCAS31* (cold acclimation-specific 31) encodes a dehydrin which acts as a positive regulator of drought response [43,44]. In the *ZmNAC20*-overexpressing lines, *Zm00001eb376710* (*ZmDHN2*), encoding a dehydrin protein showed increased transcript level compared with the wild-type B104, suggesting that ZmNAC20 promoted *ZmDHN2* expression (Figure 6). Moreover, we found that *ZmDHN2* was upregulated by ABA (Figure 7). *Zm00001eb283860* (*ZmGA2OX6*) encodes a gibberellin 2-beta-dioxygenase that may deactivate gibberellin (GA). A previous study showed that low GA activity promoted stomatal closure and enhanced drought resistance [45]. Overexpression of *ZmNAC20* increased the transcript level of *ZmGA2OX6*, suggesting that ZmNAC20 might regulate drought resistance through modulation of GA signaling via *ZmGA2OX6* (Figure 6). In addition, *ZmGA2OX6* was also upregulated by ABA, indicating that ABA participated in the expression of *ZmGA2OX6* (Figure 7). *Zm00001eb130550* (*ZmSWEET17A*) encodes a sugar transporter. SUGAR WILL EVENTUALLY BE EXPORTED TRANSPORTER (SWEET) proteins promote the transport of different sugars over cellular membranes and control both inter and intracellular distribution of sugars [46]. In *Arabidopsis thaliana*, knockout of *SWEET17* resulted in impaired drought resistance [46], and mutation of *SWEET11* and *SWEET12* also exhibited reduced drought resistance [47]. *ZmSWEET17A* was regulated by ZmNAC20 and ABA (Figure 6D and Figure 7D), suggesting that ZmNAC20 might regulate drought resistance by modulating the *ZmSWEET17A*-mediated sugar transporter. *Zm00001eb076200* (*ZmPx15*) encodes a peroxidase. The water-deficit condition caused the excessive production and accumulation of reactive oxygen species (ROS), which damaged the plant cells. Peroxidase acted as an antioxidant to scavenge ROS [48]. Overexpression of *ZmNAC20* enhanced the transcript level of *ZmPx15*, implying that ZmNAC20 might improve drought resistance by modulation of *ZmPx15* (Figure 6). ABA also promoted the expression of *ZmPx15*, suggesting that ABA regulated the expression of *ZmPx15* (Figure 7). These results suggested that ZmNAC20 regulated expressions of various genes involved in drought stress response. ZmNAC20 improved drought resistance by modulation of multiple genes, which constituted a complex network.

## 3. Discussion

Drought is among the major environmental factors that affect plant growth and crop yield [3]. To obtain stress resistance, many transcription factor genes are activated under drought conditions. The transcription factors receive the upstream drought stress signal and activate expressions of many stress-responsive genes [49]. These molecular responses help plants rapidly adapt to the water-deficit stress environment [25]. The transcription factors-mediated networks are critical and necessary for plants to survive under drought conditions [5]. It has been demonstrated that NAC transcription factors play crucial roles in drought stress response [8,13,14,17,20]. In this study, we reported a NAC transcription factor gene *ZmNAC20*, which was upregulated by drought stress (Figure 1A,B). Overexpression of *ZmNAC20* resulted in an increased survival rate and a higher level of relative water content (Figure 2B,C), suggesting that ZmNAC20 functioned as a positive factor in improving drought resistance. Previous studies have reported that several maize *ZmNACs* genes, including *ZmNAC111* [13], *ZmNAC33* [20], *ZmNAC55* [14], *ZmNAC49* [17], *ZmNST3* [15], *ZmNUT1* [21], *ZmNAC080308* [22], *ZmSNAC13* [23], *ZmNAC84* [24], and *ZmNAC48* [16], contributed to improving drought resistance. In maize, a large number of *ZmNACs* genes have been identified, but the function of most of them has remained elusive until now. Considering the important function of NACs in drought stress response, more efforts on revealing the function of *ZmNACs* genes in maize should be made in future studies.

Stomata are important structures to control water loss and gas exchange [38,50]. Under drought conditions, stomatal closure is regulated by multiple signals, such as ABA, ROS, Ca^2+^, and H_2_S, [34,38,51]. Among these signals, ABA is the center molecule to control stomatal closure in response to drought stress [25,26]. *ZmNAC20* was upregulated by drought stress and ABA (Figure 1A,B). Overexpression of *ZmNAC20* accelerated the closure of stomata in response to ABA. The *ZmNAC20*-overexpressing transgenic maize showed a smaller stomatal aperture and lost less water than the wild-type B104 (Figure 3A,C). These results suggested that ZmNAC20 contributed to promoting ABA-mediated stomatal closure in response to drought stress. ZmNAC20 partially improved drought resistance by promoting stomatal closure. In maize, ZmNAC48 contributes to improving drought resistance by promoting stomatal closure, whereas ZmNAC49 enhances drought resistance by the decline of stomatal conductance and stomatal density [16,17]. ZmNST3 increases drought resistance by activating the expression of genes related to cellulose synthesis in the secondary cell wall [15]. ZmNUT1 improves drought resistance by affecting water transportation via regulating the expression of the cellulose synthase gene and cysteine proteolytic enzyme gene [21]. These findings indicated that ZmNACs transcription factors improved drought resistance by modulation of various pathways, suggesting that the function of NACs has diversity and complexity.

Using RNA-seq analysis, we obtained a list of genes regulated by ZmNAC20 (Tables S1 and S2). GO enrichment analyses indicated that ZmNAC20 regulated multiple pathways that are mainly involved in abiotic stimulus, response to water deprivation, and response to hormones (Figure 5B). RNA-seq analysis indicated that ZmNAC20 regulated the expression of various stress-responsive genes, such as the transcription factor genes *ZmEREB106*, *ZmEREB145*, *ZmEREB188*, *ZmWRKY106*, and *ZmbZIP75*, the dehydrin gene *ZmDHN2*, the GA dioxygenase gene *ZmGA2OX6*, the sugar transporter gene *ZmSWEET17A*, and antioxidant enzyme gene *ZmPx15* (Figure 6 and Figure 7). These results suggested that ZmNAC20 improved drought resistance by modulation of multiple pathways. ZmNAC20 formed a network to cope with drought stress. The molecular response induced by ZmNAC20 promoted plants to adapt to the water-deficit environment.

There are many limitations when using transcription factors to improve drought resistance in crops. We should consider the fact that many transcription factors inhibit plant growth and development, while improving drought resistance. Stomatal closure prevents water loss and enhances drought resistance but also limits CO_2_ intake and photosynthesis [3]. For example, overexpression of *ZmNAC49* enhances drought resistance in maize, but also significantly decreases stomatal conductance and stomatal density. *ZmNAC49* overexpression affects the expression of genes related to stomatal development [17]. Overexpression of *ZmNAC48* improves drought resistance by enhancing stomatal closure [16]. Less studies focus on revealing the mechanism of plants maintaining growth under drought conditions. It is necessary to identify the genes that increase drought resistance but do not limit plant growth and development. Previously, a study reported that overexpression of a wheat ABA receptor gene *TaPYL4* improves drought resistance by reducing stomatal conductance but does not affect wheat growth and development. This is because overexpression of *TaPYL4* increases water use efficiency and enhances photosynthesis [52]. Future studies should pay more attention to those transcription factors that can improve drought resistance without penalizing plant growth. In this study, we did not detect whether ZmNAC20 affected water use efficiency and photosynthesis. Further research will be focused on revealing the function of ZmNAC20 to maintain growth under drought conditions.

Taken together, our work revealed the function of ZmNAC20 in drought stress response in maize. The study provides valuable insights into the role of ZmNAC20 in promoting drought resistance and shed light on the molecular mechanisms underlying plant adaptation to water-deficit stress.

## 4. Materials and Methods

### 4.1. Plant Materials and Growth Conditions

The overexpression transgenic plants in this study were derived from the maize (*Zea mays* L.) inbred line B104, which is a public, transformable maize inbred line and has been widely used in recent studies [15,53,54,55]. To analyze the function of ZmNAC20 in drought stress response, the coding sequence of ZmNAC20 was cloned and inserted into a modified pCambia3300 vector. *ZmUbiquitin1* (Zm*Ubi*) was used as the promoter to drive *ZmNAC20* expression. The *ZmNAC20-OE* construct was transformed into the maize inbred B104. Seeds of the maize inbred line B104 and *ZmNAC20*-overexpressing transgenic lines (*ZmNAC20-OE1* and *ZmNAC20-OE2*) with the same size were selected, and immersed in 75% ethanol solution for disinfection for 1 min. After disinfection, the maize seeds were washed with distilled water 5–6 times. Then, the seeds were immersed in the distilled water and cultivated in the dark at 26 °C for 24 h. The soaked seeds with embryo sides faced upward were placed evenly in the seedling tray with filter paper, adding a proper amount of distilled water (the seeds just touched the water surface). After that, the seedling tray was sealed with plastic wrap, and placed in darkness at 28 °C for 3 days. The germinated seeds were transplanted into the mixed nutrient soil (vermiculite: nutrient soil = 1:1) and cultivated in the 28 °C plant light incubator under a photoperiod of 16 h/8 h (day/night), 60% relative humidity, and 16,800 Lux light intensity. After three true leaves grew from the maize seedlings, the seedlings were subjected to the follow-up experiment.

### 4.2. Drought Treatment

When maize seedlings of B104, *ZmNAC20-OE1*, and *ZmNAC20-OE2* developed three true leaves, water was withheld for drought stress treatment. After about 15 days of drought treatment, watering was resumed to allow the plants to recover. The survival rate was recorded after three days of rewatering. Seedlings with green leaves and hard stems were recorded as survivors. In each test, at least 20 plants for each line were involved. Phenotype and data analysis was based on three independent experiments.

### 4.3. Relative Water Content Assay

After about 7 days of drought treatment, the middle segments of fresh leaves were cut about 5 cm long. The weight of the middle segments of fresh leaves was measured immediately and recorded as the fresh weight. Then the above leaves were immersed in distilled water at room temperature for 12 h. After that, the saturated fresh weight was recorded. At last, the leaves were placed in a 70 °C oven for 12 h, and the dry weight was measured. We used the formula [relative water content= (fresh weight − dry weight)/(saturated fresh weight − dry weight) × 100%] to calculate the relative water content of leaves. Statistical analysis was based on three independent experiments.

### 4.4. Detached Leaves Water Loss Assay

The above-ground parts of B104 and *ZmNAC20-OE1* maize seedlings with three true leaves were cut and spread in the plant light incubator, with a temperature of 28 °C, relative humidity of 30%, and light intensity of 16,800 Lux. The dry weight (DW) of detached leaves was measured at 15 min, 45 min, 60 min, 100 min, 120 min, 160 min, 180 min, 200 min, 300 min, and 400 min after dehydration stress treatment. The weight of detached leaves was measured as fresh weight (FW) at 0 min of dehydration stress treatment. We used the formula [relative water loss rate = (FW − DW)/FW × 100%] to calculate the relative water loss rate. Statistical analysis was based on three independent experiments.

### 4.5. Determination of Biomass

After drought stress treatment for about 7 days, the aerial parts of maize seedlings were cut off and immediately weighed. The weight was recorded as fresh weight. The aerial parts of maize seedlings were then put into a 70 °C oven for continuous drying for 24 hours. After that, the weight of the above materials was measured as the dry weight. Biomass (g) = dry weight or fresh weight. There were three biological replicates in total, with every replicate containing four maize seedlings.

### 4.6. Stomatal Aperture Assay

The aerial parts of B104 and *ZmNAC20-OE1* maize seedlings with three true leaves were cut off and immersed in the stomatal opening buffer (10 mM KCl, 50 mM CaCl_2_, and 10 mM MES/Tris, pH 5.6), and then incubated in dark for 2 hours and in light for 3 hours. After incubation in the stomatal opening buffer, the leaves were taken out and immediately put into the 0, 1, and 10 μM ABA solution (taking stomatal opening buffer as a solution and ABA as solute) prepared in advance respectively. At 1 h of ABA treatment, transparent nail polish was coated on the upper epidermis of maize leaves. When nail polish was completely dried, the upper epidermis was torn off with tweezers and made into a temporary slide. Ten stomata were randomly selected in the view of the microscope, and the length and width were measured with Image. Three biological replicated assays were performed.

### 4.7. Subcellular Localization Assay

To determine the subcellular localization of ZmNAC20, the coding sequence without the stop code of *ZmNAC20* was cloned and inserted into a *35S:GFP* vector, obtaining *35S:NAC20-GFP*. *35S:H2B-mCherry* is a nuclear localization marker. The constructs of *35S:ZmNAC20-GFP* and *35S:H2B-mCherry* were transformed into the *Agrobacterium tumefaciens* strain GV3101, then used for transient expression in leaves of tobacco (*Nicotiana benthamiana*). The constructs *35S:GFP* and *35S:H2B-mCherry* were used to act as the control. The *A. tumefaciens* GV3101 harboring *35S:ZmNAC20-GFP* and *35S:H2B-mCherry* or *35S:GFP* and *35S:H2B-mCherry*, were cultured overnight at 28 °C and centrifuged for 10 min at 5000 rpm. The cultures were resuspended using the buffer solution with 10 mM MES (pH 5.5), and 10 mM MgCl_2_·6H_2_O. The cultures were then infiltrated into the leaves of about one-month tobacco seedlings. After being cultured for 3 days, the infiltrated leaves were imaged using a Nikon A1 HD25 confocal microscope. The merged signals of GFP and RFP showed a yellow color.

### 4.8. qRT-PCR Assay

Total RNA from leaves was extracted using RNA-Solv^®^ Reagent of Omega Bio-tek (No. R6830). The RNA was reverse-transcribed to synthesize cDNA by TIANGEN’s FastKing RT Kit (With gDNase) (No. KR116). The resultant cDNA was used as a template for the detection of relative gene expression, with the mazie *ZmUbiquitin* gene as the reference gene. qRT-PCR was performed with Hieff^®^qPCR SYBR^®^Green Master Mix of YEASEN (11201ES08). Each gene had three technical repeats and three biological repeats. Sequences of qRT-PCR primers were given in Table S3.

### 4.9. RNA-seq and Data Analysis

Three biological repeats of the wild type (WT-R1, WT-R2, and WT-R3) and the *ZmNAC20*-overexpressing transgenic plants *ZmNAC20*-*OE1* (OE-R1, OE-R2, and OE-R3) were used for RNA sequencing (RNA-seq). Total RNAs were isolated from the detached leaves which were treated with dehydration for 3 h using TRI reagent (Sigma; catalog no. T9424, Sigma-Aldrich, Burlington, MA, USA). RNA was sequenced using Illumina 2500 instrument in Berry Genomics, Beijing, China. The clean reads were aligned to the genome sequences of maize (Zm-B73-REFERENCE-NAM-5.0) that was downloaded from the website (plants.ensembl.org); HISAT2 software (version 2.0.5) was used for the clean reads [56]. The gene expression levels were measured using StringTie software (version 1.3.6) [56]. Differential gene expression (DEG) analysis was conducted using DESeq2 software (version 1.34.0) [57]. DEG was screened by using the cutoff of the log_2_ |fold change| ≥ 1 and *q*-value (adjusted *p*-value) ≤ 0.05 between the *ZmNAC20*-*OE1* and the wild-type B104. The analysis of Gene Ontology (GO) enrichment was performed using AgriGO v2.0, a web-based tool, and a database for GO analyses [58]. The pathway KEGG enrichment for the DEG was performed using the OmicShare tools, a free online platform for data analysis (www.omicshare.com/tools, accessed on 7 February 2023). The RNA-seq data have been deposited into the sequence read archive (SRA) database of NCBI under accession number PRJNA893876.

## 5. Conclusions

In this study, we found a nucleus-localized NAC transcription factor ZmNAC20 that improved drought resistance in maize. *ZmNAC20* was rapidly induced by drought and ABA. Overexpression of *ZmNAC20* promoted stomatal closure and prevented water loss. ZmNAC20 regulated multiple pathways by modulation of many stress-responsive genes expression. Our results indicated that ZmNAC20 functioned as a positive regulator to improve drought resistance in maize. ZmNAC20 contributed to improving drought resistance by modulation of stomatal closure and activation expression of stress-responsive genes.

## Data Availability

Not applicable.

## Supplementary Materials

The supporting information can be downloaded at: https://www.mdpi.com/article/10.3390/ijms24054712/s1.
