# Supplementary figures and images for: Transcription Factor ZmNAC20 Improves Drought Resistance by Promoting Stomatal Closure and Activating Expression of Stress-Responsive Genes in Maize
Source: Int J Mol Sci. 2023 Mar 1;24(5):4712. doi: 10.3390/ijms24054712 (PMC10003513; doi:10.3390/ijms24054712)

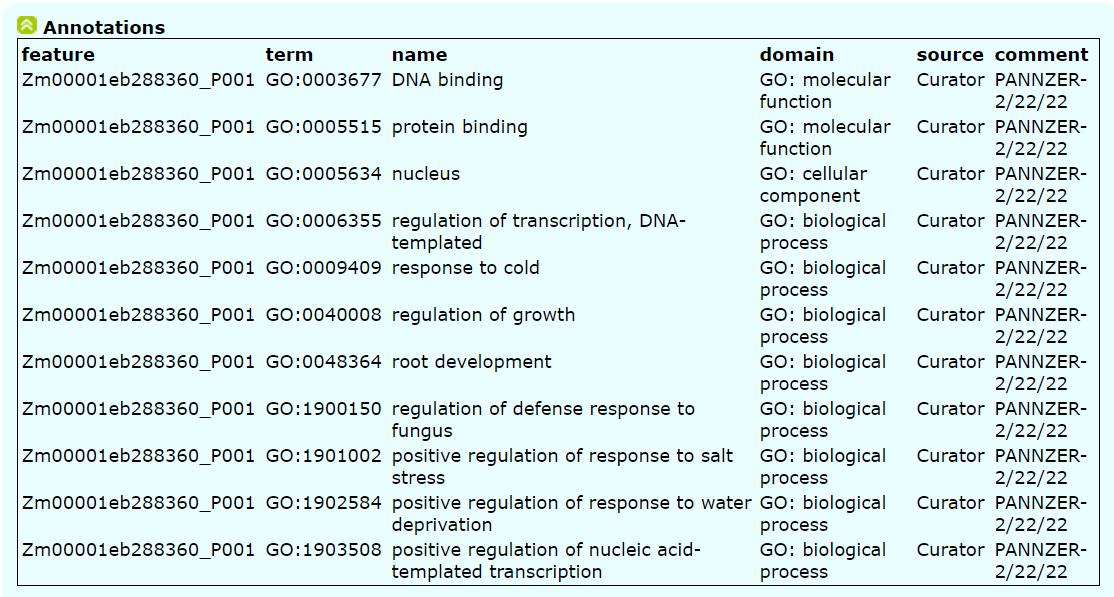

Supplement: Supplementary file 1 [file ijms-24-04712-s001.zip › Figure S1.tif]

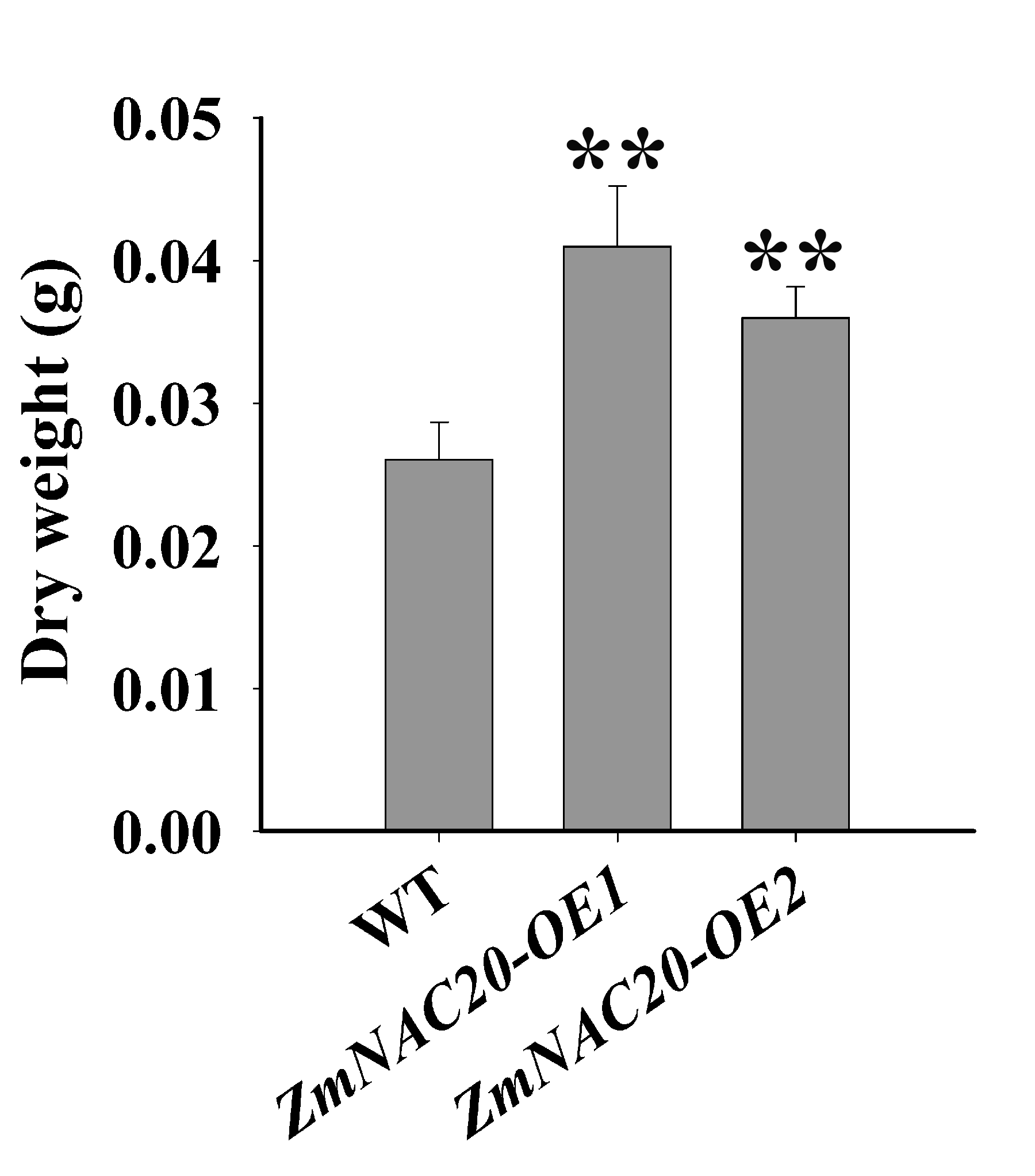

Supplement: Supplementary file 1 [file ijms-24-04712-s001.zip › Figure S2.tif]

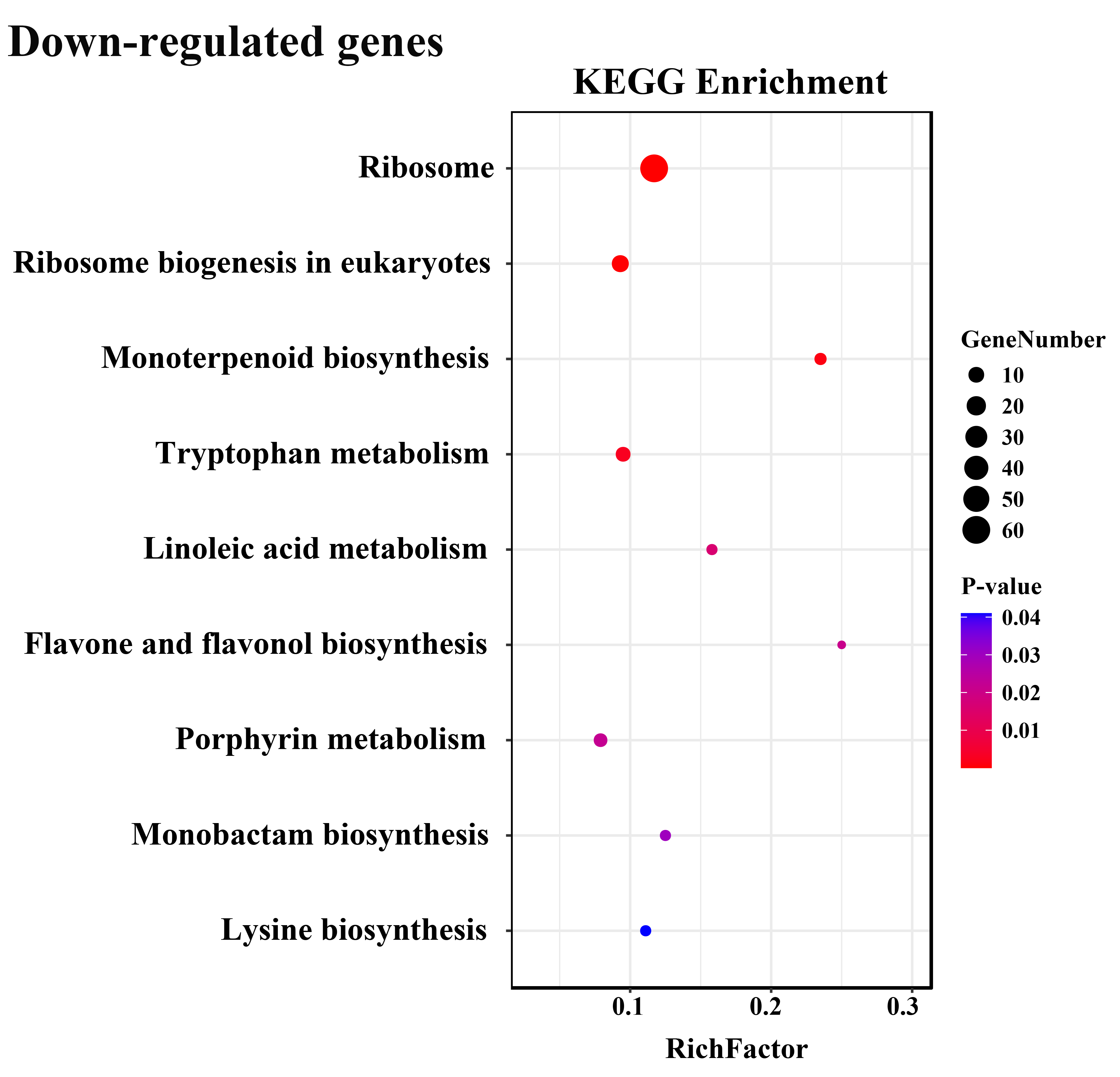

Supplement: Supplementary file 1 [file ijms-24-04712-s001.zip › Figure S4.tif]
